# Retrotransposon-centered analysis of piRNA targeting shows a shift from active to passive retrotransposon transcription in developing mouse testes

**DOI:** 10.1186/1471-2164-12-440

**Published:** 2011-09-01

**Authors:** Tobias Mourier

**Affiliations:** 1 Centre for GeoGenetics, Natural History Museum, University of Copenhagen, Øster Voldgade 5-7, DK-1350 Copenhagen K, Denmark

## Abstract

**Background:**

Piwi-associated RNAs (piRNAs) bind transcripts from retrotransposable elements (RTE) in mouse germline cells and seemingly act as guides for genomic methylation, thereby repressing the activity of RTEs. It is currently unknown if and how Piwi proteins distinguish RTE transcripts from other cellular RNAs. During germline development, the main target of piRNAs switch between different types of RTEs. Using the piRNA targeting of RTEs as an indicator of RTE activity, and considering the entire population of genomic RTE loci along with their age and location, this study aims at further elucidating the dynamics of RTE activity during mouse germline development.

**Results:**

Due to the inherent sequence redundancy between RTE loci, assigning piRNA targeting to specific loci is problematic. This limits the analysis, although certain features of piRNA targeting of RTE loci are apparent. As expected, young RTEs display a much higher level of piRNA targeting than old RTEs. Further, irrespective of age, RTE loci near protein-coding coding genes are targeted to a greater extent than RTE loci far from genes. During development, a shift in piRNA targeting is observed, with a clear increase in the relative piRNA targeting of RTEs residing within boundaries of protein-coding gene transcripts.

**Conclusions:**

Reanalyzing published piRNA sequences and taking into account the features of individual RTE loci provide novel insight into the activity of RTEs during development. The obtained results are consistent with some degree of proportionality between what transcripts become substrates for Piwi protein complexes and the level by which the transcripts are present in the cell. A transition from active transcription of RTEs to passive co-transcription of RTE sequences residing within protein-coding transcripts appears to take place in postnatal development. Hence, the previously reported increase in piRNA targeting of SINEs in postnatal testis development does not necessitate widespread active transcription of SINEs, but may simply be explained by the prevalence of SINEs residing in introns.

## Background

Retrotransposable elements (RTE) constitute a significant proportion of mammalian genomes. The RTEs proliferate through an RNA stage that is subsequently reverse transcribed back to genomic DNA [1]. The high level of divergence in RTE insertions between closely related organisms [2-5] and the link between RTE insertions and diseases [6-8] witness the ongoing activity of RTEs in mammalian genomes. Several genomic mechanisms are devised to minimize the proliferation of RTEs acting both at pre- and post-transcriptional levels [9-11].

### Mouse retrotransposable elements

Around 40 percent of the mouse genome consists of RTE sequence, slightly lower than observed for the human genome, although this presumably is a result of the higher substitution rate in mouse, limiting the identification of old RTE sequence [12,13]. RTEs are divided according to the presence or absence of long terminal repeats (LTR). Mammalian LTR elements consist mainly of endogenous retroviruses (ERV) that at some point during evolution have been inserted in the germline and fixed. Although the amount of sequence occupied by LTR elements is comparable between human and mouse, the level of *de novo *mutations caused by LTR element activity is extensively higher in mouse than in human [8,14]. The most abundant ERV class in the mouse genome (~5.5%) is the Class III ERVs, which in the RepeatMasker [15] annotation - upon which this study is based - is broadly divided in two groups, the ERVL and MaLR elements. The latter is a non-autonomous transposon, meaning that the elements do not encode the enzymatic machinery required for its own transposition. The Class II ERVs (~4% of the genome), annotated as ERVK in RepeatMasker, is believed to be younger than Class III ERVs [16] and consists of a broad range of clades, including the IAP elements (Intracisternal A-type Particles). Class I ERVs (ERV1 in RepeatMasker) cover less than 1% of the mouse genome. Through ectopic recombination between the flanking LTR sequences, solitary LTR sequences may be formed. In RepeatMasker, terminal LTR sequences and the internal sequences (residing between the terminal LTRs in a complete LTR retrotransposon) are annotated independently. Although the terminal and internal sequences may in many cases be determined to form a single LTR retrotransposon, for simplicity, the two annotations (termed 'LTRter' and 'LTRint', respectively) are analysed independently in this study.

Non-LTR retrotransposons are divided into LINEs (Long INterspersed Elements) that are autonomous, and SINEs (Short INterspersed Elements) that are non-autonomous. LINEs occupy roughly 20% of the mouse genome. The majority of mouse LINE elements belong to the L1 superfamily, which contains sub-families that are still active [17-19]. Despite the comparable levels of genome occupied by LINE sequences in human and mouse [12,13], there are more than 15 times as many full-length L1 elements with intact open reading frames in the mouse genome [20]. Almost 1.5 million SINE elements are present in the mouse genome, making up approximately 8% of the total genome size. Unlike the human genome where a single SINE, the Alus, is dominating [21], the mouse genome harbours two successful superfamilies of SINEs, Alu and B2 that are present in equal numbers [12]. The evolutionary histories of the mouse SINEs are truly different; Alus are derived from a 7SL RNA, whereas B2s evolved from a tRNA sequence [21,22].

### Piwi proteins and small RNAs

Piwi-associated RNAs (piRNAs) are small (24-30 nucleotides long) RNAs that bind Piwi proteins of the Argonaute family [23,24]. The mouse genome encodes 3 Piwi proteins, MILI, MIWI and MIWI2 that all binds piRNAs in the male germline [25,26]. Initially, piRNAs from adult mouse testis were found to contain less RTE sequence than would be expected from the genomic content of RTEs, suggesting that piRNAs were not specifically targeting RTEs [27,28]. However, a later study on piRNAs from an earlier (pre-pachytene) stage showed a high content of RTE sequence in piRNAs [29]. Further evidence for the involvement of mouse piRNAs in controlling RTE activity came with the finding that knockout of Mili and Miwi2 resulted in reduced piRNA levels and increased RTE transcription [29,30]. Knockout mice further showed decreased DNA methylation levels at RTE loci [31,32]. As the temporal expression of Piwi proteins in developing mouse testis coincides with the resetting of genomic methylation [33], it is hypothesised that piRNAs act as guides for the methylation machinery [29,31,32].

By analysing the piRNAs bound to MIWI2 and MILI, Aravin and colleagues [31] suggested the following scenario: In prenatal development (16.5 days postcoitum, dpc), transcripts from full-length active RTEs are the main substrates for piRNAs that primarily associate with MILI (and to a lesser extent to MIWI2). Available transcripts containing antisense RTE sequence bind this complex and antisense RTE piRNAs are formed which in turn associate primarily with MIWI2 (and MILI, respectively). Both complexes may bind complementary RTE transcripts, entering the so-called ping-pong amplification cycle of piRNAs, in which Piwi-bound piRNAs pair with complementary transcripts that are subsequently cut into new piRNAs having a 10 nucleotide overlap with the template piRNAs [31,34]. In prenatal development, piRNAs are primarily targeting L1 and IAP RTEs, for which activity has been reported at this stage [35,36]. In postnatal development (10 days postpartum, dpp) MIWI2 is no longer detectable, whereas MILI is present throughout germline development [31,37,38]. The overall level of piRNA targeting of RTE sequences drops at 10 dpp, but interestingly, a relative increase in the piRNAs targeting B1 SINEs (members of the Alu superfamily) was observed [31].

This raises two fundamental questions. Firstly, do Piwi proteins discriminate between transcripts and how is RTE sequences then distinguished from other transcripts? The finding of piRNAs targeting supported a scenario with limited discrimination [31]. Secondly, what lies behind the apparent shift in RTEs being targeted by piRNAs during development in male mouse germline? By analysing to extent to which genomic RTE loci are targeted by piRNAs in developing mouse testes, the present study aims at assessing the transcriptional dynamics of RTE during development, and consider the relationship between RTE activity and piRNA generation further.

The data for such analysis should meet a range of criteria. Although numerous mouse RNA libraries are available, only a small subset is derived from wild-type developing mouse testes [39]. Further, as the prevalent transcription of RTEs will results in a large population of fragmented transcripts of sizes similar to piRNAs, analysis should be restricted to libraries of RNAs associated with Piwi proteins. This limits the available data to libraries from the above-mentioned study by Aravin and colleagues [31].

## Results and Discussion

### Theoretical piRNA coverage of individual RTE loci

Three libraries of small RNAs bound to MIWI2 and MILI proteins in mouse male germline [31] were reanalysed; one library with MIWI2-bound RNAs from 16.5 dpc (henceforth referred to as 'MIWI2 early') and two libraries with MILI-bound RNAs from 16.5 dpc and 10 dpp ('MILI early' and 'MILI late', respectively) (Table 1).

**Table 1:** Piwi-RNA sequence read libraries

					Percent of mapped reads covering:			
					
Library	Stage		Raw reads (× 1000)	Mapped reads (× 1000)	LINE (19.6)	SINE (7.6)	LTRint (3.0)	LTRter (7.4)
MIWI2 early	16.5 dpc	prenatal	1,940	1,934	30.2	5.2	34.8	17.0

MILI early	16.5 dpc	prenatal	472	470	19.2	5.3	16.3	11.9

MILI late	10 dpp	postnatal	1,327	1,324	7.7	19.9	15.9	13.3

Three piRNA libraries from reference [31] were used for this study (accession numbers: MIWI2 early, GSM319957; MILI early, GSM319956; MILI late, GSM319953). Mapped reads denote the number of RNA reads mapping perfectly at least once to the mouse genome. The percentage of mapped reads mapping to loci of annotated RTEs are shown to the right. In parentheses under the RTE type the fractions of the mouse genome (mm9) occupied by the RTE sequences according to the RepeatMasker [15] annotation at the UCSC Genome Browser [67] are shown.

**Figure 1:**
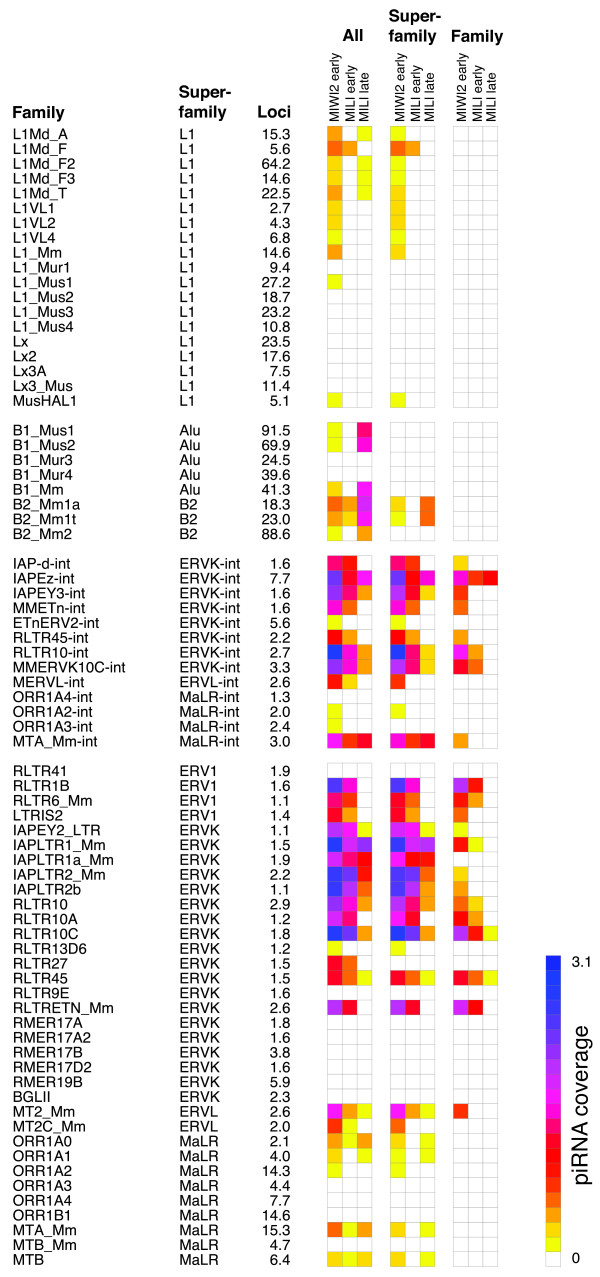
**Median piRNA coverage of RTE loci**. Theoretical piRNA coverage levels (see Methods sections) are shown as colours indicating the median log2 values for all loci of belonging to a given RTE family. Only RTE families with at least a 1000 annotated loci and a median value above zero in any library are shown (albeit very low levels are not visible in this representation). For each RTE family, the superfamily it belongs to and the number of loci (in thousands) is listed. Families of internal LTR sequences are suffixed by '-int' in their superfamily. Three sets of columns are shown (All/Superfamily/Family), each set containing the three libraries (MIWI2 early, MILI early and MILI late). The left column set (All) shows coverage of all mapped piRNA reads. The middle column set (Superfamily) shows coverage of piRNAs exclusively mapping to this superfamily of RTE, and the right column set (Family) the coverage of piRNAs exclusively mapping to the particular RTE family.

To analyse in detail the theoretical piRNA coverage of each individual RTE loci, reads from each RNA library were mapped onto the mouse genome. Only perfectly mapping reads were considered. Due to the inherent redundancy of RTE sequences, reads mapping uniquely to RTE loci are scarce and therefore all mapping reads were considered. The number of reads mapping to each RTE loci was weighted by the uniqueness of the reads, so that the count from each read was simply divided by the number of times that particular read mapped to the entire genome. Further, read coverage was weighted by library size and length of the locus, in an attempt to allow direct comparisons between libraries and RTE types. As seen from Figure 1, differences in this theoretical piRNA coverage differ markedly between RTE families and between libraries. When only considering reads that are exclusively mapping within a particular superfamily of RTEs (*e.g*. L1 LINEs) the overall patterns of coverage still remain (middle column in Figure 1). For LTR elements (both internal sequence and terminal repeats), most major trends are still observable when only considering reads mapping entirely within a single family of RTE (*e.g*. internal sequence of IAP-d elements) (right column in Figure 1). Unless otherwise noted, in the following all reads are used for analysis, and only RTE families with a least a thousand genomic members are considered (see Additional File 1, Table S1 for a list of these 318 RTE families). The previously reported decrease in piRNA coverage of IAP LTRs and L1 LINEs and the corresponding increase in SINE coverage during development [31] are clearly evident from Figure 1 (left column). Also consistent with earlier findings [31], the median MILI early piRNA coverage of individual RTE families is highly correlated with the median coverage from MIWI2 early piRNAs, but not with the coverage from MILI late piRNAs (Additional File 1, Figure S1).

### Higher piRNA coverage of younger elements

**Figure 2:**
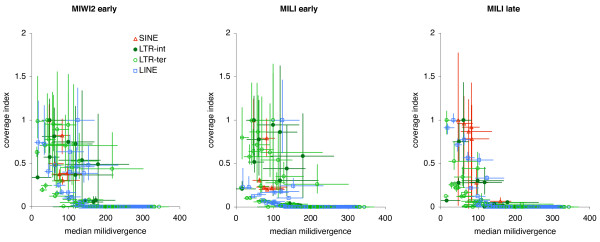
**RTE age and piRNA coverage**. The median millidivergence from consensus (as a proxy for age) is plotted against the average piRNA coverage (all reads) values. RTE families are coloured according to their type as indicated on the left chart. To allow for easier comparison between RTE types, coverage values are indexed so that the family with the highest average coverage is set to a value of 1. Error bars denote 25 and 75 percentiles for both piRNA coverage and millidivergence.

The RepBase database of repeated sequences [40] contain consensus sequences for individual RTE families, and RepeatMasker [15] annotation is based on sequence similarity to these consensus sequences. As the vast majority of RTE loci are under no negative selection (but see, for example [41-43]) the level of divergence between genomic loci and the RepBase consensus sequence can be taken as a proxy for the age of the RTE family. When plotting median piRNA coverage of RTE families against their median divergence, a clear trend of highly covered RTEs being relatively young is observed irrespective of RTE type (Figure 2). For all types of RTEs, the average piRNA coverage of younger elements is significantly higher than coverage of older elements (Additional File 1, Table S2). Also, the high level of variation in piRNA coverage between individual RTE loci is evident from the percentiles shown in Figure 2.

### Gene expression levels and piRNA coverage

**Figure 3:**
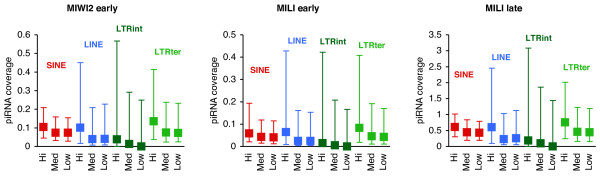
**Expression levels and piRNA coverage around genes**. Genes are divided according to their expression levels in adult testis (high/medium/low) as indicated below charts. The median piRNA coverage of RTEs around genes are shown as rectangles with error bars denoting 25 and 75 percentiles. Coverage values are normalised by the number of base pairs occupied by RTEs around the genes. Using a Mann-Whitney U test, all values for highly expressed genes are significantly higher than the corresponding values for lowly expressed genes for all four RTE types. Significance levels shown in Additional File 1, Table S3.

To test the piRNA coverage in the genomic context of protein-coding genes, all known genes with at least 20 kb (kilo base pairs) to the nearest neighbouring gene (in both directions) and with available Affymetrix expression data from testis tissue were retrieved [44]. These 3307 genes were grouped into highly expressed genes (25% highest expression signals, 827 genes), lowly expressed genes (25% lowest, 829 genes), and medium expressed genes (rest, 1651 genes). For each gene set, the piRNA coverage of RTE residing within 10 kb upstream of their annotated transcription start sites to within 10 kb of their stop sites was recorded (Figure 3). For all types of RTE elements, piRNA coverage in the context of highly expressed genes was significantly higher than both medium and lowly expressed genes. This was found for all piRNA libraries (Additional File 1, Table S3).

For some RTE types, the relative number of young and old loci differ between the vicinities of highly expressed genes and lowly expressed genes, (Additional File 1, Figure S2), suggesting that the higher levels of piRNA coverage of RTE near highly expressed genes could simply be explained by the age of the RTE sequences. However, when repeating the analysis without the youngest RTE sequences, essentially similar results and significance levels are found (Additional File 1, Table S3).

Interestingly, when assessing the piRNA coverage of RTE sequence near transcription start sites (TSS), peaks are observed immediately upstream of TSS on the reverse strand, and for piRNAs not targeting RTEs, also immediately downstream of TSS on the forward strand (Additional File 1, Figure S3). Such a pattern resembles that of the recently discovered short transcripts generated around TSS (the TSS-associated RNAs) [45,46] suggesting that these piRNAs may in fact be TSS-associated RNAs. It is uncertain if this represents experimental contamination of non-piRNAs or if these TSS-associated RNAs provide the transcripts that a processed into piRNAs, although the presence of RNA reads smaller than the usual 24-30 nucleotides - especially among early MILI piRNAs - hints that a contribution from the former scenario cannot be ruled out (Additional File 1, Figure S4). Assuming all RNAs mapping within 1000 base pairs of an annotated TSS are TSS-associated RNAs and removing these from the analysis does not change any of the presented conclusions (data not shown).

### piRNA coverage and distance to genes

**Figure 4:**
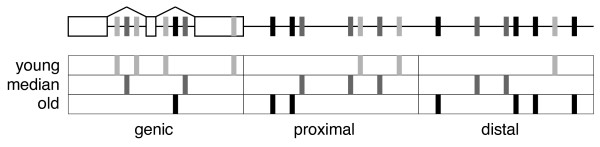
**Grouping of RTE loci**. A schematic overview of the procedure used to group RTE loci within each family. A hypothetical genome is shown on top with a single protein-coding gene (exons denoted by boxes). RTE loci are shown as vertical lines, with age indicated by increasing colour darkness. The RTE loci are first divided in three equally sized groups (rows below genome) based on age, then divided according to their genomic location (columns below genome). The border between proximal and distal loci is set to the median of the distances between all non-genic loci and the nearest gene. Within each age group, the location groups can now be directly compared against each other. Note that the numbers of RTEs in age groups are equal by definition, whereas this may not be the case in the location groups.

The age of RTEs and their genomic distance to protein-coding genes is not independent [13,47]. If RTEs residing near genes are in general relatively young, one would expect these RTEs to display high levels of piRNA coverage as a result of this. To test if proximity to genes affected piRNA coverage independent of RTE age, members of each RTE family were divided into three equally sized groups based on their divergence from their consensus sequence (called 'young', 'median' and 'old' loci, respectively). Within each age-group, members were further divided into sub-groups according to genomic location; i) RTE loci residing inside the boundaries of known genes (termed 'genic'), ii) RTE loci in intergenic regions in proximity to known genes ('proximal'), and iii) RTE loci in intergenic regions distal to known genes ('distal'). The groups 'proximal' and 'distal' defined as loci closer or further away from genes, respectively, than the median distance of all non-'genic' loci from the RTE family. An overview of the grouping procedure is presented in Figure 4. The assumption that younger RTE members tend to reside closer to genes are confirmed by the observation that for 95% of all RTE families, the fraction of loci being proximal to genes is higher for young loci than for old loci (Wilcoxon Signed Rank, p < 2.2 × 10^-16^; values not shown).

**Figure 5:**
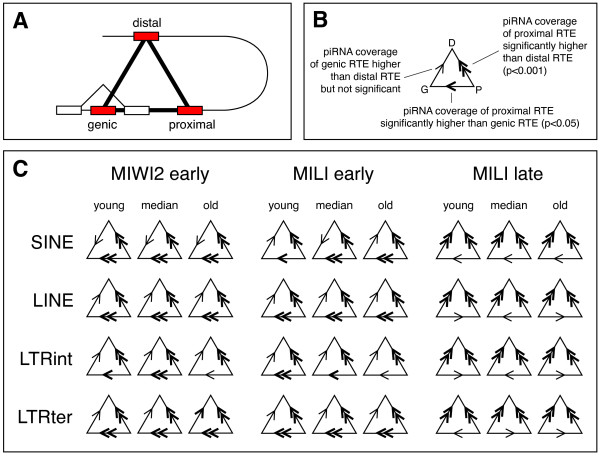
**piRNA coverage and genomic context**. A) Schematic depiction of the 3 categories of RTE loci (shown as red boxes). Genic RTEs reside inside the boundaries of protein-coding genes (exons shown as white boxes), proximal RTEs reside close to genes, and distal RTEs reside far from genes. The three RTE boxes are connected by a triangle, and in the remainder of the figure this triangle will symbolise the three categories of RTE loci. B) Example of the triangle graphic. The corners of the triangle correspond to the three categories of RTE loci (highlighted by their first letter in this example), and larger-than and smaller-than signs denote the relative levels of piRNA coverage of the categories. One thin sign corresponds to a non-significant difference, one bold sign to a significant difference at the 0.05 level, and two bold signs to a significant difference at the 0.001 level (see Methods section). C) Symbolic presentations of the real differences in piRNA coverage levels. RTEs are grouped into the 4 shown types (left), and differences are shown for the 3 libraries (top), with RTE loci divided according to age (for each family independently, see main text for details). Absolute values and significance levels are available in Additional File 1, Table S4.

**Figure 6:**
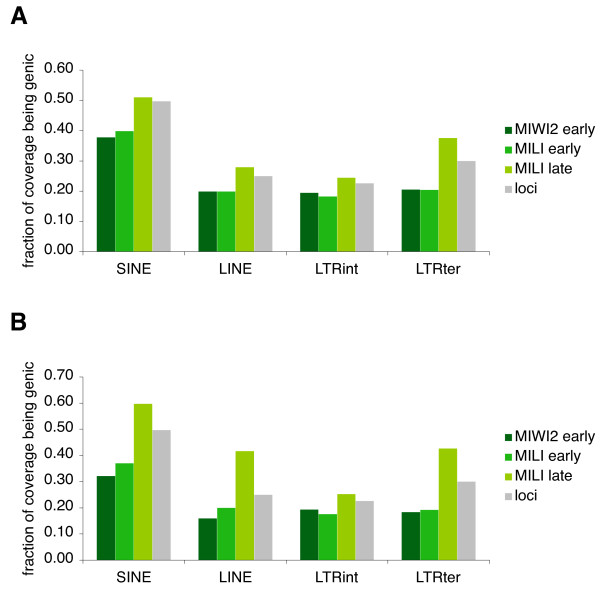
**Proportion of piRNA coverage targeting genic RTE loci**. The fractions of total piRNA coverage that are mapped to genic loci are shown for the four RTE types. Green bars denote different read libraries as indicated on the right. Gray bars show the proportion of loci residing in a genic context. Values are shown for A) All reads and B) Reads mapping exclusively within a single RTE family.

RTE loci proximal to genes have - irrespective of age - significantly higher piRNA coverage than similar RTE loci distal to genes (Figure 5). With internal LTR sequences belonging to the 'old' group as the only exception, loci proximal to genes have significantly higher coverage than genic loci in prenatal development. Interestingly, in postnatal development no RTE group displays a significantly higher coverage for proximal loci than for genic loci, and furthermore, for all RTE groups, genic loci have significantly higher coverage than loci distal to genes (Figure 5). Thus, coverage by MILI late piRNAs is enriched in genic regions, an observation that is further supported by the fact that the total coverage of MILI late piRNAs mapping to genic regions is increased for all types of RTEs (Figure 6).

### Strand bias in piRNA coverage of genic RTEs

**Figure 7:**
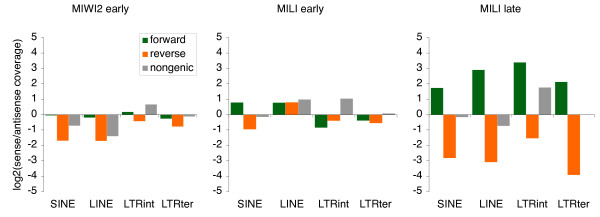
**Strand bias**. The log2 ratio of sense and antisense coverage from uniquely mapping piRNAs shown for the 4 main RTE types. Each RTE type is divided into genic loci on the forward strand relative to the host gene ('forward'; green columns), genic loci on the reverse strand ('reverse'; orange columns), and loci residing outside genes ('nongenic'; grey columns). All values are shown for the three piRNA libraries.

Aravin and colleagues [31] showed that in early development, the substrate for piRNA generation is provided by actively transcribed RTE elements. Later in development, active transcription of RTEs should then be repressed, and mRNA sequences from active full-length RTE loci are no longer widespread. This suggests that co-transcription of RTE sequences along with mRNAs from protein-coding genes (predominantly in intronic regions) could now take on a relatively larger role in providing RTE sequence transcripts. A prerequisite for the generation of piRNAs is the presence of transcripts with complementary sequences. Although active RTEs need to be transcribed from their forward strand, the RTE sequences scattered around the highly transcribed genome could produce transcripts in both orientations. But if as suggested, transcribed RTE sequences in postnatal mouse testes are mainly provided from co-transcription with genes, the transcriptional orientation of a given genic RTE loci is to a large extent determined by the orientation of the host gene. One can therefore test if the strand of piRNAs mapping uniquely to genic RTE loci corresponds to the orientation of the RTE relative to the host gene. Of course, amplification from the ping-pong cycle may generate multiple piRNAs, which may map on both strands of a genic RTE locus (although the efficiency of the ping-pong cycle may decrease in postnatal development as MIWI2 is no longer expressed [31]), potentially blurring the picture. As seen from Figure 7, a clear pattern of high sense coverage of genic RTEs in the forward orientation and high antisense coverage of genic RTEs in the reverse orientation is seen for postnatal piRNAs, but not for prenatal piRNAs.

## Conclusions

As reported previously, RTE families are targeted very differently by piRNAs in developing mouse testes. By focusing on the total population of RTE loci, the present reanalysis of published data reveal further differences in piRNA targeting between individual members of RTE families. The available data for this analysis is arguably limited and the presented data relies on a single set of experiments. Although deep-sequencing techniques ideally should provide sequences from all available transcripts in a neutral fashion, biases may be introduced experimentally, especially during construction of libraries [48]. Furthermore, considerable biological differences in RTE sequences have been reported between mouse strains [49,50]. Nevertheless, the vast majority of RTE sequences will be shared among all extant mice, and the results presented here are all of a global genomic character with no predictions for individual loci, suggesting a fair generality of the findings.

Transcriptional activity is correlated between genomic regions residing near each other [51-53], and the observation that piRNA targeting of RTEs is higher around highly expressed genes, may simply reflect that transcription of RTEs is more permissible near highly expressed genes. A correlation between transcription levels of LTR sequences and their neighbouring genes has previously been reported in fission yeast [54]. This further supports the notion that RTE transcripts are not specifically recognized as RTEs by the Piwi proteins, but are largely triggering the piRNA response in a manner proportional to their presence. It should be stressed that the reported preference by MILI for sense RTE sequences and the corresponding preference by MIWI2 for antisense sequences [31] suggest some level of discrimination of transcripts.

In postnatal testis development, piRNA targeting is shifted towards loci residing in introns of protein-coding genes. If, as assumed, active transcription of RTE loci is repressed at this stage, one would expect a higher proportion of RTE sequences in the total transcriptome to be derived from co-transcription of intronic RTE loci. This observation could at least in part explain the previously observed increase in piRNAs targeting SINE elements in postnatal stages [31], as SINE elements are the most abundant RTEs in introns (Additional File 1, Figure S5). Therefore, the increased piRNA response directed at SINE sequences does not necessitate transcription of active SINE elements in postnatal development. In fact, as SINE elements are non-autonomous, presumably using the enzymatic machinery provided by LINE elements [55,56], there should be no basis for SINE proliferation in postnatal development if the prenatal silencing of LINE persists. Yet, SINE transcription may take place without subsequent transposition, and the known functional effects of mammalian SINE transcription [57-59] and the recently reported SINE RNA toxicity [60] suggest both active SINE transcription in later development, and the possible need for regulation.

On an evolutionary time scale, RTE activity has contributed hugely to the evolution of mammalian genomes [61-64], and when attempting to understand the diversity of present eukaryotic life it is essential to include the history and activity of RTEs. However, RTEs are not just silent passengers that occasionally spring into action, but have to be dealt with within each individual's life history. In this respect, the indirect approach of analysing small RNAs generated to repress RTE activity in the germline may produce further valuable knowledge on the activity of RTEs during development.

## Methods

### Data and Annotations

Small RNA libraries accession numbers GSM319953 (MILI late), GSM319956 (MILI early) and GSM319957 (MIWI2 early) were retrieved through DeepBase (http://deepbase.sysu.edu.cn/) [39] and mapped to the mouse genome (mm9 assembly) using bowtie [65]. Prior to mapping, reads were filtered and sequences with ambiguous base calls and low-complexity sequences were removed. The latter was done measuring the linguistic complexity [66] of the sequences in 16 bp windows, and excluding reads with an average complexity of less than 0.75. Preliminary tests showed that this would remove highly repetitive reads with very large numbers of genomic mappings (not shown). For each library, this procedure filtered out between 0.14 and 0.17% of all raw reads. RepeatMasker and known gene annotations were downloaded from the UCSC Genome Browser [15,40,67]. A set of non-overlapping TSS was selected by grouping all known genes according to their assignment to ENSEMBL genes [68]. For each ENSEMBL gene, the most abundant genomic start point was selected. If more than one point was found to have the highest abundance, the one furthest upstream of these was chosen. Gene expression levels were assessed from the 'testis' signal intensities in the Mouse GNF1M Gene Atlas from BioGPS (http://biogps.gnf.org/) [44].

### Mapping and coverage

For each RTE loci the number of reads mapped within the locus were recorded. Reads were assigned a score of 1/(number of genomic mappings of read), so that only uniquely mapping reads scored 1. The read score were then divided by the size of the RTE loci (in kilo-base pairs). Finally, scores were divided by the total number (in millions) of mapped reads from the library in question.

### Statistical testing

To test for difference between RTE loci from different genomic regions (data presented in Figure 5), all RTE families were first split in 3 groups based on age where after members in each group were divided according to their genomic context (genic, proximal, distal). Hence, for each RTE family, nine sets of loci were formed, and the average piRNA coverage for each set was recorded. To test for difference between two groups (for example, between old LINE loci being genic or distal), pairs of average values were collected for the 90 LINE families (Additional File 1, Table S1) and tested using a Wilcoxon Signed-Rank Test. Bonferroni corrections (n = 108 in Figure 5) were calculated as: p_corrected _= 1-(1-p)^n^. All statistical analyses were carried out using R [69].

## Competing interests

The authors declare that they have no competing interests.

## Authors' contributions

TM conceived of the study, analyzed the data and wrote the manuscript.

## Supplementary Material

Additional file 1 **Supplementary Material**. Figures S1-S5 and Tables S1-S4. PDF format. Click here for file
