# Supplementary material for: Retrotransposon-centered analysis of piRNA targeting shows a shift from active to passive retrotransposon transcription in developing mouse testes
Source: BMC Genomics. 2011 Sep 1;12:440. doi: 10.1186/1471-2164-12-440 (PMC3175481; doi:10.1186/1471-2164-12-440)
Supplement: Additional file 1 — Supplementary Material. Figures S1-S5 and Tables S1-S4. PDF format. [file 1471-2164-12-440-S1.PDF]

Supplementary Material

Retrotransposon-Centered Analysis of piRNA Targeting Supports a Shift from Active to Passive Retrotransposon Transcription in Developing Mouse Testes

Supplementary Figure Legends

Figure S1 - Correlation between piRNA coverage from different libraries

Scatter plots of the median log2 piRNA coverage from Figure 1 in main text are shown between the three RNA libraries. Correlation coefficients are shown on each plot.

Figure S2 - Age of RTEs near genes

Each type of RTE is divided into young, median and old members. For a set of genes divided into three categories according to expression levels in adult testis, the percentage of RTEs in the vicinity of these genes belonging to the different RTE age classes is shown.

Figure S3 - piRNA Coverage around Transcription Start Sites

(A) Position-specific piRNA coverage was recorded for 10k upstream and 10k downstream of 7798 TSS, which did not overlap similar 20k context of any TSS from another annotated gene. For each 1k bin around these TSS the piRNA coverage of TEs were recorded for each strand separately. Coverage on the forward strand relative to the gene shown as positive values, coverage on the reverse strand as negative values. Dark red lines denote the 25% highest expressed genes, orange lines all genes, and yellow lines the 25% lowest expressed genes. A remarkable trend of higher coverage immediately before the TSS on the reverse strand is observed, and furthermore, this trend is more pronounced for highly expressed genes (left charts). Coverage of piRNA not mapping to RTE loci reveals an additional peak immediately

after TSS on the forward strand (right charts). The lack of this piRNA peak in TE coverage may be explained by the scarcity of TEs in this region (see figure S5B). To smoothen the curves, the individual bins (from one specific gene) with the 1% highest coverage are removed from the plots. (B) Genes and bins as in (A). Average number of bases in each bin taken up by RTE loci. Red lines=SINE, Blue lines=LINE, Green lines=LTR (LTRint and LTRter combined).

Figure S4 - Size of RNA reads around TSS

The distribution of read sizes shown for reads mapping within 1000 bp upstream of an annotated transcription start site (TSS) on the reverse strand relative to the gene (blue circles), reads mapping within 1000 bp downstream of TSS on the forward strand (red squares). For comparison, the size distributions for all other reads mapping within 10.000 bp of TSS are shown (black lines).

Figure S5 - Number of Genic RTE loci

For 318 RTE families, the percentage of loci residing within boundaries of annotated genes (genic RTE loci) plotted against the total number of loci (top). Below is a plot of the absolute number of genic loci against the total number of loci.

Figure S1

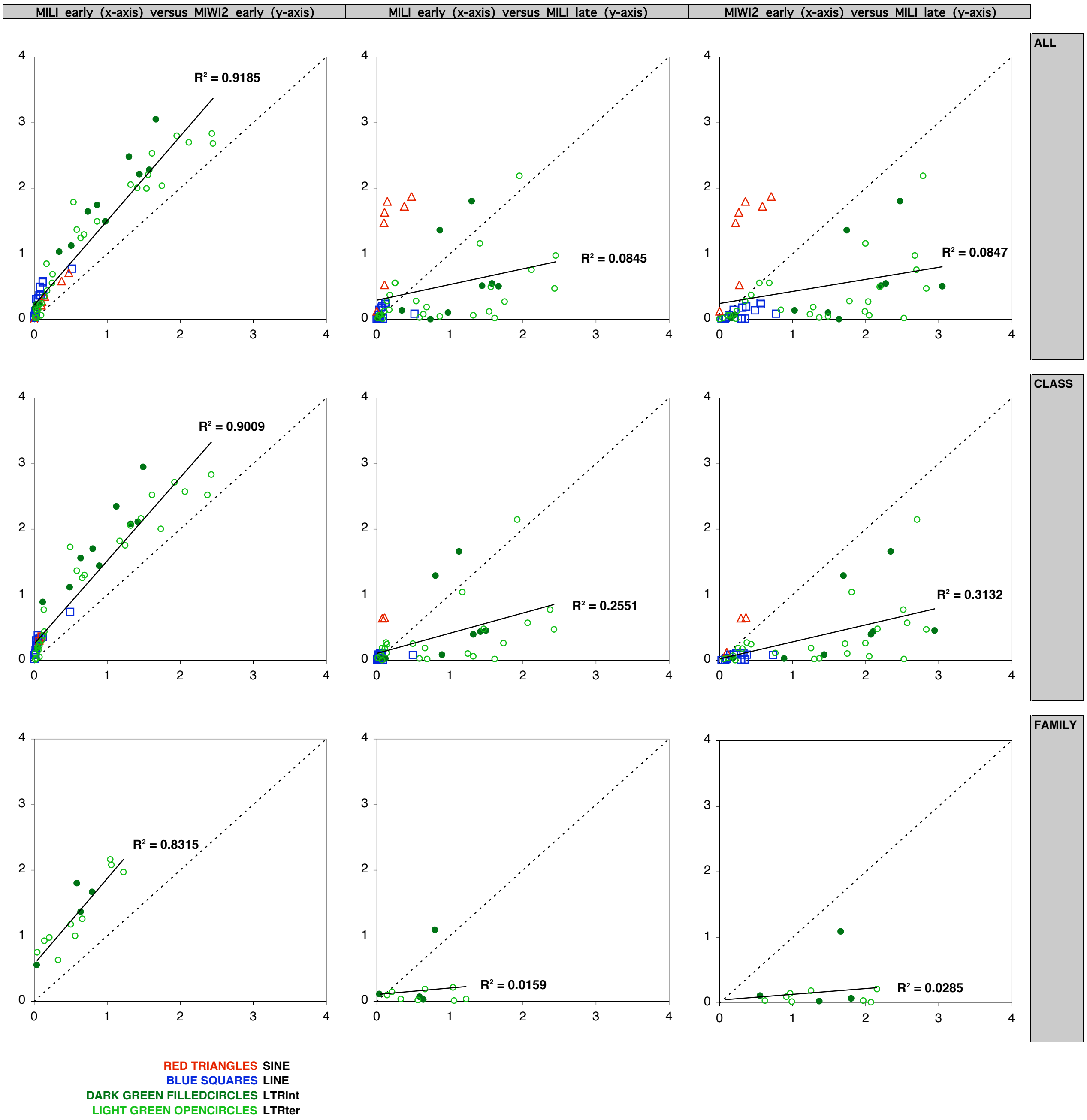
MILI early (x-axis) versus MIWI2 early (y-axis)
MILI early (x-axis) versus MILI late (y-axis)
MIWI2 early (x-axis) versus MILI late (y-axis)
ALL
CLASS
FAMILY
R² = 0.9185
R² = 0.0845
R² = 0.0847
R² = 0.9009
R² = 0.2551
R² = 0.3132
R² = 0.8315
R² = 0.0159
R² = 0.0285
RED TRIANGLES SINE
BLUE SQUARES LINE
DARK GREEN FILLEDCIRCLES LTRint
LIGHT GREEN OPENCIRCLES LTRter

Figure S2

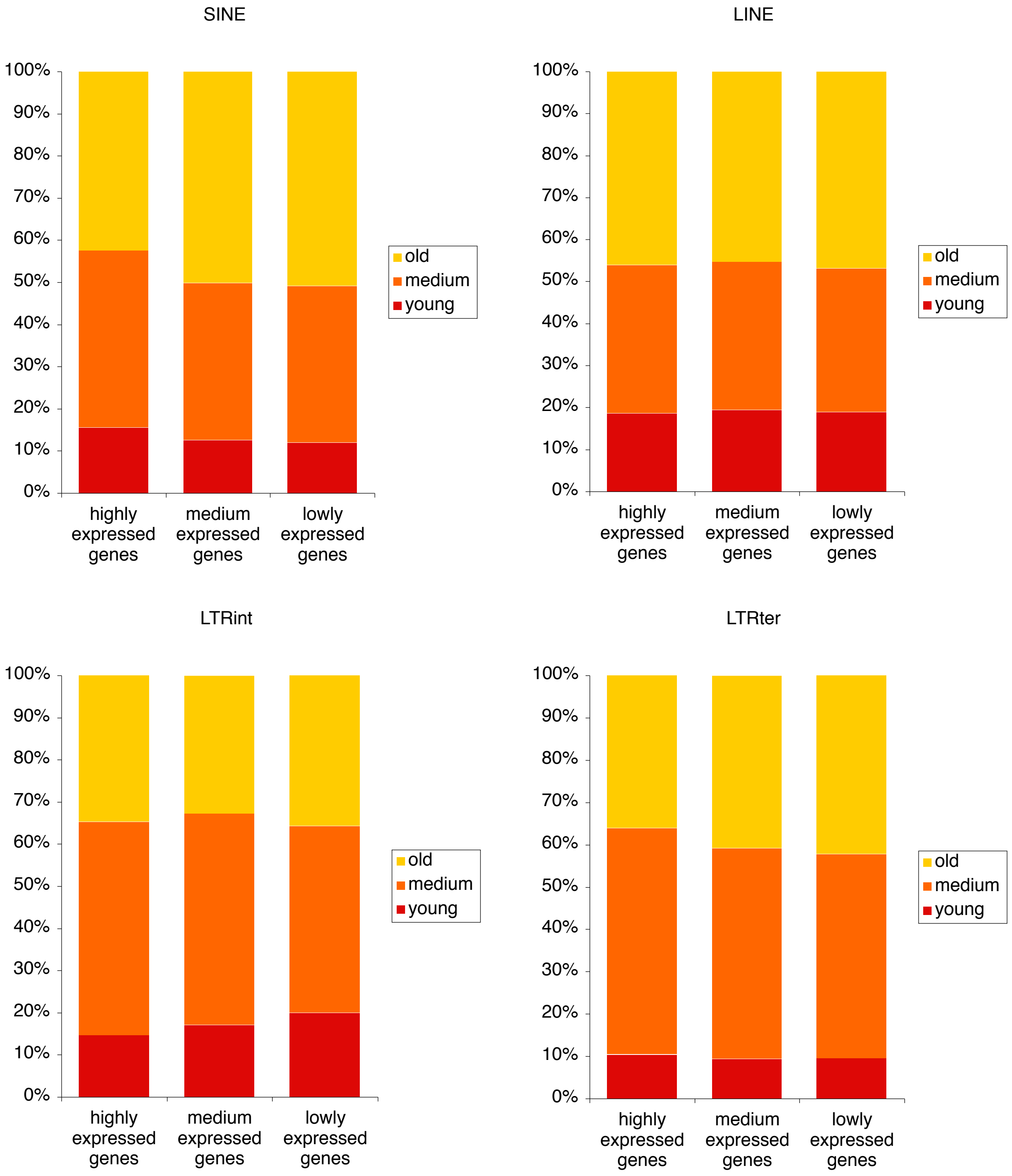
SINE
LINE
LTRint
LTRter
100%
90%
80%
70%
60%
50%
40%
30%
20%
10%
0%
old
medium
young
highly expressed genes
medium expressed genes
lowly expressed genes

Figure S3a

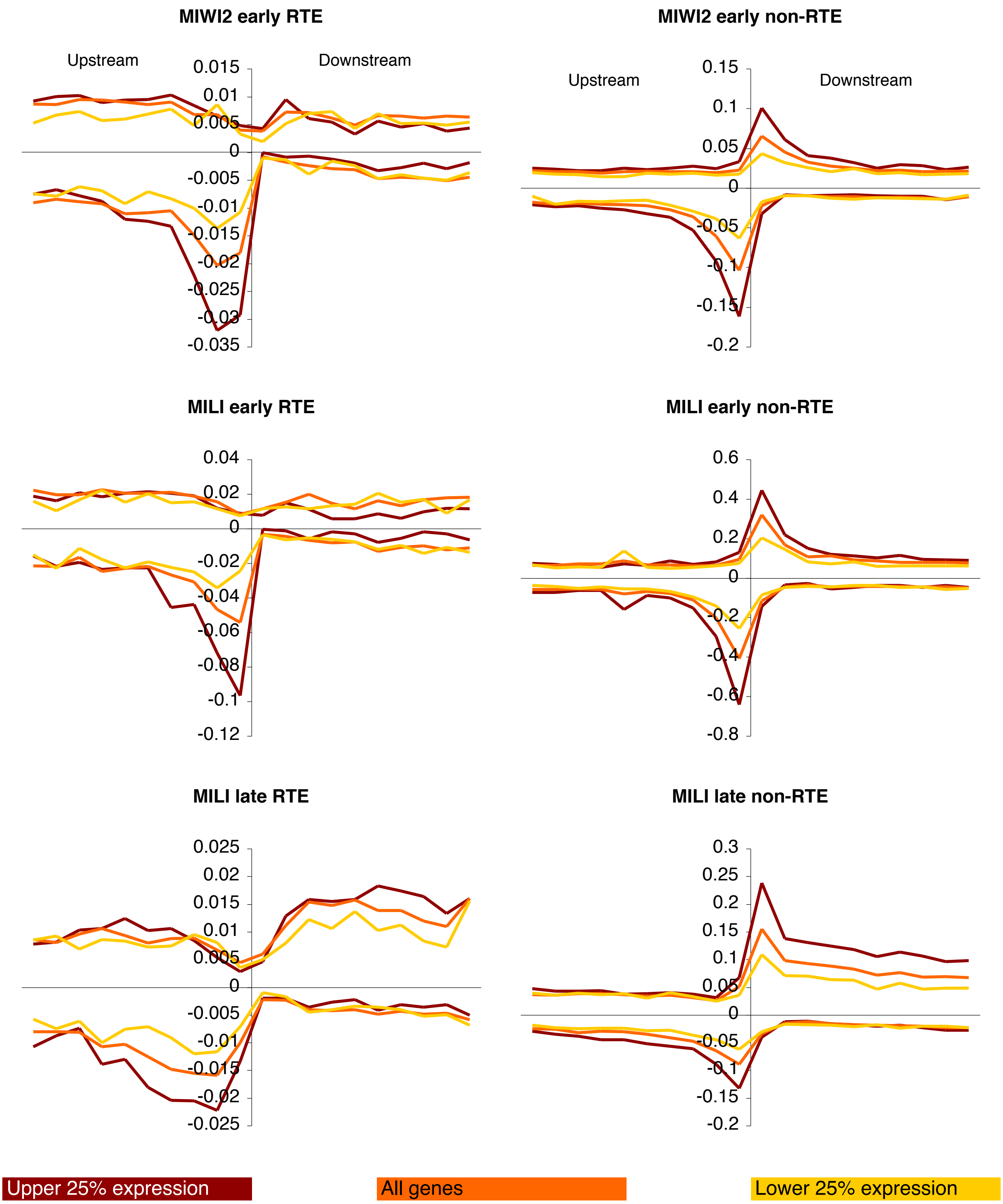
MIWI2 early RTE
Upstream
Downstream
0.015
0.01
0.005
0
-0.005
-0.01
-0.015
-0.02
-0.025
-0.03
-0.035
MIWI2 early non-RTE
Upstream
Downstream
0.15
0.1
0.05
0
-0.05
-0.1
-0.15
-0.2
MILI early RTE
0.04
0.02
0
-0.02
-0.04
-0.06
-0.08
-0.1
-0.12
MILI early non-RTE
0.6
0.4
0.2
0
-0.2
-0.4
-0.6
-0.8
MILI late RTE
0.025
0.02
0.015
0.01
0.005
0
-0.005
-0.01
-0.015
-0.02
-0.025
MILI late non-RTE
0.3
0.25
0.2
0.15
0.1
0.05
0
-0.05
-0.1
-0.15
-0.2
Upper 25% expression
All genes
Lower 25% expression

Figure S3b

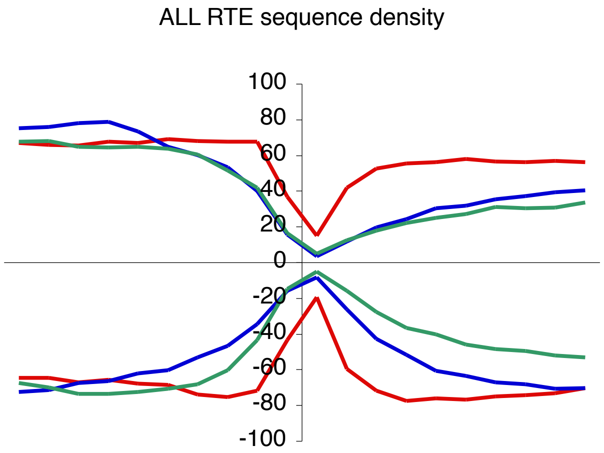

Figure S4

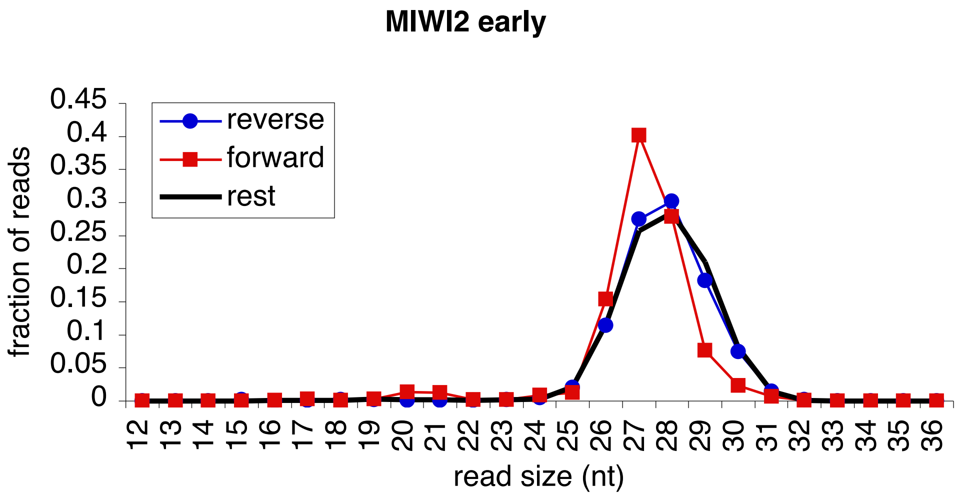
MIWI2 early
reverse
forward
rest
fraction of reads
0
0.05
0.1
0.15
0.2
0.25
0.3
0.35
0.4
0.45
12 13 14 15 16 17 18 19 20 21 22 23 24 25 26 27 28 29 30 31 32 33 34 35 36
read size (nt)

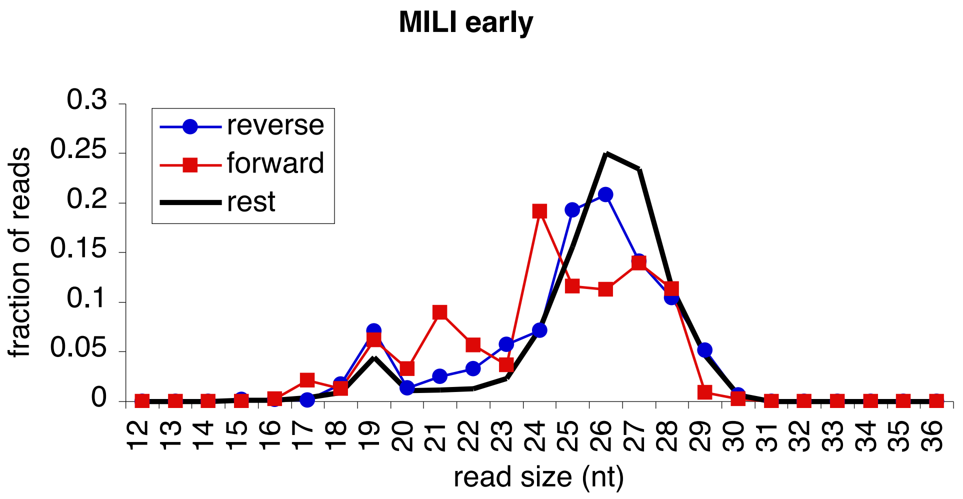
MILI early
reverse
forward
rest
fraction of reads
0
0.05
0.1
0.15
0.2
0.25
0.3
12 13 14 15 16 17 18 19 20 21 22 23 24 25 26 27 28 29 30 31 32 33 34 35 36
read size (nt)

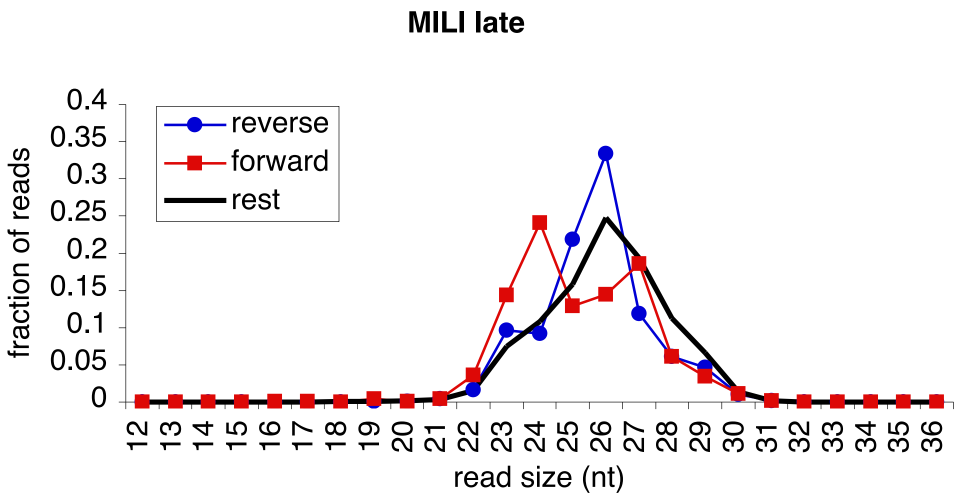
MILI late
reverse
forward
rest
fraction of reads
0
0.05
0.1
0.15
0.2
0.25
0.3
0.35
0.4
12 13 14 15 16 17 18 19 20 21 22 23 24 25 26 27 28 29 30 31 32 33 34 35 36
read size (nt)

Figure S5

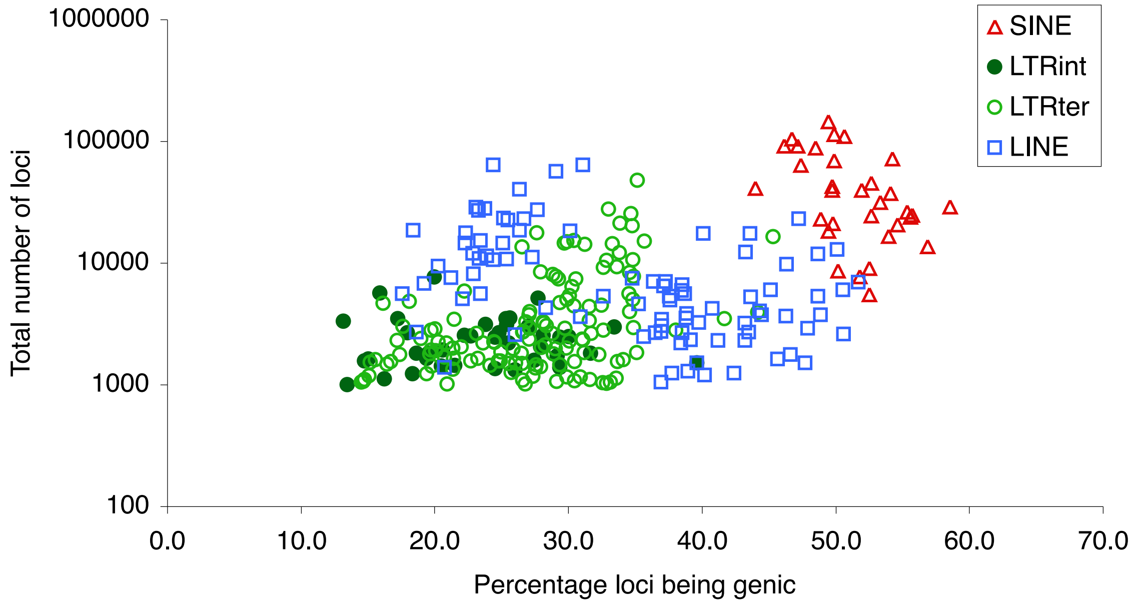

SINE
LTRint
LTRter
LINE
1000000
100000
10000
1000
100
Total number of loci
0.0
10.0
20.0
30.0
40.0
50.0
60.0
70.0
Percentage loci being genic

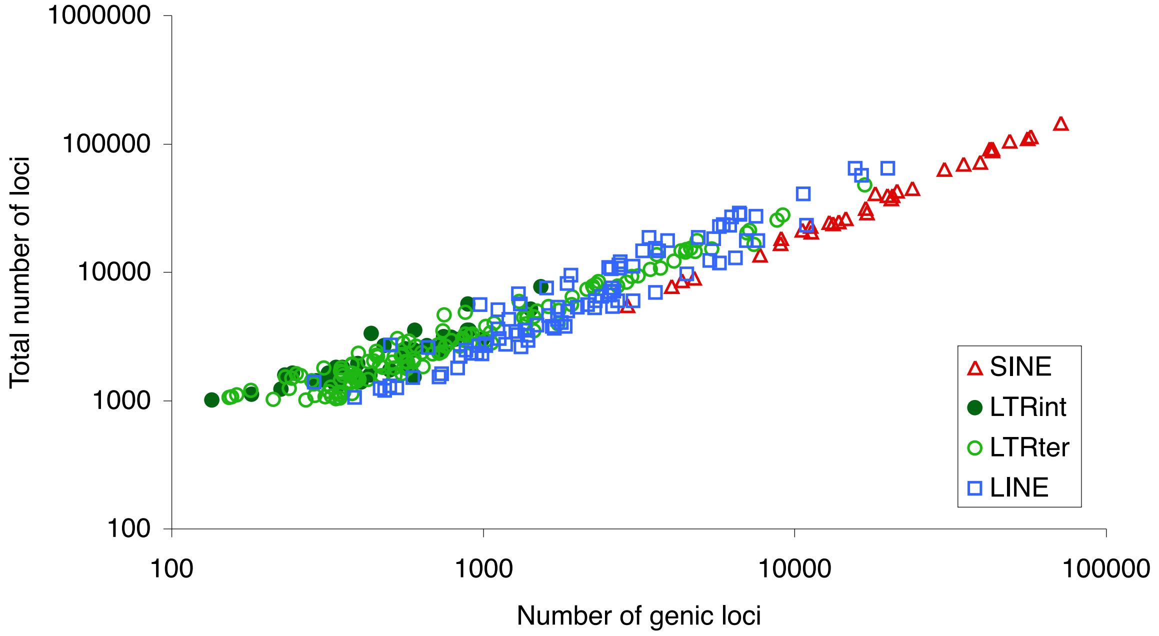

SINE
LTRint
LTRter
LINE
1000000
100000
10000
1000
100
Total number of loci
100
1000
10000
100000
Number of genic loci

Table S1

SINE

	Superfamily	Loci	base pair (x1000)
B1_Mm	Alu	41277	5470
B1_Mur1	Alu	37635	4662
B1_Mur2	Alu	31615	3888
B1_Mur3	Alu	24545	3069
B1_Mur4	Alu	39585	4882
B1_Mus1	Alu	91451	12284
B1_Mus2	Alu	69938	9025
B1F	Alu	45267	4995
B1F1	Alu	20681	2251
B1F2	Alu	16689	1856
PB1	Alu	13632	1318
PB1D10	Alu	72485	6915
PB1D7	Alu	24899	2417
PB1D9	Alu	29145	2848
B2_Mm1a	B2	18276	3099
B2_Mm1t	B2	22966	3793
B2_Mm2	B2	88624	13696
B3	B2	144766	24391
B3A	B2	91510	13761
B4	B4	63694	10781
B4A	B4	104751	19230
ID_B1	B4	110572	14575
RSINE1	B4	114638	14331
ID	ID	7777	471
ID2	ID	5530	398
ID4	ID	23905	1716
ID4_	ID	26352	1871
MIR	MIR	42872	5293
MIR3	MIR	9034	998
MIRb	MIR	39780	5162
MIRc	MIR	21261	2282
MIRm	MIR	8649	778

LINE

	Superfamily	Loci	base pair (x1000)
HAL1	L1	3760	859
HAL1-2a_MD	L1	1292	201
HAL1-3A_ME	L1	6597	598
L1_Mm	L1	14565	9172
L1_Mur1	L1	9432	6321
L1_Mur2	L1	18541	10014
L1_Mur3	L1	26886	12540
L1_Mus1	L1	27181	22221
L1_Mus2	L1	18665	14131
L1_Mus3	L1	23161	17787
L1_Mus4	L1	10750	7964
L1_Rod	L1	11120	4187
L1M2	L1	18231	5409
L1M3	L1	7052	1725
L1M4	L1	12360	2947
L1M4b	L1	2750	721
L1M4c	L1	3046	905
L1M5	L1	17502	3725
L1MA4	L1	7468	2186
L1MA4A	L1	2659	781
L1MA5	L1	4972	1301
L1MA5A	L1	1240	412
L1MA6	L1	7018	1946
L1MA7	L1	3246	865
L1MA8	L1	3836	1049
L1MA9	L1	4597	1237
L1MB1	L1	2472	664
L1MB2	L1	2915	765
L1MB3	L1	5351	1383
L1MB4	L1	2716	683
L1MB5	L1	3761	984
L1MB7	L1	6924	1874
L1MB8	L1	5993	1582
L1MC	L1	6467	1465
L1MC1	L1	5597	1684
L1MC2	L1	2195	628
L1MC3	L1	5325	1555
L1MC4	L1	5989	1360
L1MC4a	L1	3654	862
L1MC5	L1	2616	525
L1MCa	L1	3616	992
L1MD	L1	5961	1377
L1Md_A	L1	15286	30804
L1Md_F	L1	5564	8623
L1Md_F2	L1	64168	71381
L1Md_F3	L1	14569	13126
L1Md_T	L1	22506	46480
L1MD1	L1	2298	680
L1MD2	L1	3210	837

LINE(cont.)

	Superfamily	Loci	base pair (x1000)
L1MD3	L1	2338	519
L1MDa	L1	3446	935
L1ME1	L1	5234	1460
L1ME2	L1	2657	723
L1ME2z	L1	1513	349
L1ME3	L1	1248	294
L1ME3A	L1	2318	556
L1ME3B	L1	1504	371
L1ME4a	L1	1777	376
L1MEc	L1	5347	1442
L1MEd	L1	1048	222
L1MEe	L1	1201	275
L1MEf	L1	3507	897
L1MEg	L1	4255	942
L1VL1	L1	2707	2318
L1VL2	L1	4263	4711
L1VL4	L1	6754	7495
Lx	L1	23481	15578
Lx2	L1	17571	10364
Lx2A	L1	1384	845
Lx2A1	L1	2568	1330
Lx2B	L1	12049	7473
Lx3_Mus	L1	11405	8250
Lx3A	L1	7543	5091
Lx3B	L1	5613	3174
Lx3C	L1	8147	5245
Lx4A	L1	10600	5998
Lx4B	L1	10871	6040
Lx5	L1	28042	16686
Lx6	L1	28711	13638
Lx7	L1	40409	16607
Lx8	L1	63988	22271
Lx9	L1	56470	18368
MusHAL1	L1	5053	3023
L2	L2	17494	3439
L2a	L2	23133	3872
L2b	L2	12864	1615
L2c	L2	11785	1487
L3	CR1	9716	1213
L3b	CR1	1614	193
L4	RTE	4023	493

LTRint

	Superfamily	Loci	base pair (x1000)
MuRRS4-int	ERV1-int	1814	1080
RLTR14-int	ERV1-int	1818	799
RLTR6-int	ERV1-int	1226	2077
RodERV21-int	ERV1-int	1812	1056
ETnERV2-int	ERVK-int	5627	4098
IAP-d-int	ERVK-int	1567	1167
IAPEY3-int	ERVK-int	1622	1636
IAPEz-int	ERVK-int	7666	11707
MERVK26-int	ERVK-int	1003	633
MMERVK10C-int	ERVK-int	3318	2843
MMETn-int	ERVK-int	1638	2130
MurERV4-int	ERVK-int	1517	870
MYSERV16_I-int	ERVK-int	2503	1433
MYSERV6-int	ERVK-int	3135	1725
MYSERV-int	ERVK-int	1433	650
RLTR10-int	ERVK-int	2678	1923
RLTR19-int	ERVK-int	2528	815
RLTR42-int	ERVK-int	2452	658
RLTR45-int	ERVK-int	2195	1322
RMER16-int	ERVK-int	3498	1942
RMER17C-int	ERVK-int	1925	743
RMER3D-int	ERVK-int	2661	1196
ERVL-B4-int	ERVL-int	1419	457
ERVL-E-int	ERVL-int	1114	278
MERVL_2A-int	ERVL-int	3524	2016
MERVL-int	ERVL-int	2555	4230
RMER15-int	ERVL-int	3107	1133
MTA_Mm-int	MaLR-int	3004	2738
MTC-int	MaLR-int	3505	1499
MTD-int	MaLR-int	2478	1138
MTEa-int	MaLR-int	1380	641
MTE-int	MaLR-int	5127	1819
ORR1A2-int	MaLR-int	2032	1687
ORR1A3-int	MaLR-int	2439	773
ORR1A4-int	MaLR-int	1315	1024
ORR1B1-int	MaLR-int	2982	1382
ORR1D1-int	MaLR-int	1721	804
ORR1D2-int	MaLR-int	1361	574
ORR1E-int	MaLR-int	1581	718

LTRter

	Superfamily	Loci	base pair (x1000)
LTRIS2	ERV1-ter	1424	655
MER21B	ERV1-ter	3484	889
RLTR14	ERV1-ter	2804	673
RLTR1B	ERV1-ter	1555	615
RLTR23	ERV1-ter	3021	801
RLTR24	ERV1-ter	1441	428
RLTR41	ERV1-ter	1890	580
RLTR6_Mm	ERV1-ter	1097	423
RMER2	ERV1-ter	2318	827
RMER21B	ERV1-ter	1262	291
RMER5	ERV1-ter	7363	2070
BGLII	ERVK-ter	2265	791
BGLII_B	ERVK-ter	1768	591
IAPEY2_LTR	ERVK-ter	1055	340
IAPLTR1_Mm	ERVK-ter	1492	550
IAPLTR1a_Mm	ERVK-ter	1923	649
IAPLTR2_Mm	ERVK-ter	2247	1013
IAPLTR2b	ERVK-ter	1063	329
RLTR10	ERVK-ter	2884	1093
RLTR10A	ERVK-ter	1191	360
RLTR10C	ERVK-ter	1780	743
RLTR11A	ERVK-ter	3036	1047
RLTR11A2	ERVK-ter	3340	1181
RLTR11B	ERVK-ter	1978	587
RLTR12B	ERVK-ter	2921	921
RLTR13D6	ERVK-ter	1232	782
RLTR15	ERVK-ter	5398	1391
RLTR16	ERVK-ter	2354	706
RLTR17	ERVK-ter	2693	1129
RLTR18	ERVK-ter	1837	673
RLTR18B	ERVK-ter	2368	718
RLTR19	ERVK-ter	1012	304
RLTR20A	ERVK-ter	1086	298
RLTR20B3	ERVK-ter	2232	729
RLTR20C	ERVK-ter	1406	343
RLTR20D	ERVK-ter	1449	343
RLTR21	ERVK-ter	2726	995
RLTR22_Mur	ERVK-ter	3268	1431
RLTR25A	ERVK-ter	3211	1149
RLTR25B	ERVK-ter	4486	1205
RLTR26	ERVK-ter	2032	808
RLTR27	ERVK-ter	1528	237
RLTR31_Mm	ERVK-ter	1173	484
RLTR31A_Mm	ERVK-ter	1009	385
RLTR33	ERVK-ter	1573	571
RLTR40	ERVK-ter	2646	901
RLTR45	ERVK-ter	1478	621
RLTR9A	ERVK-ter	1777	522
RLTR9E	ERVK-ter	1613	551
RLTRETN_Mm	ERVK-ter	2637	829
RMER12	ERVK-ter	5578	1743
RMER12B	ERVK-ter	1038	426
RMER13A	ERVK-ter	2915	1322
RMER13B	ERVK-ter	2191	1200
RMER16	ERVK-ter	1998	630
RMER17A	ERVK-ter	1800	864
RMER17A2	ERVK-ter	1636	921
RMER17B	ERVK-ter	3766	2091
RMER17C	ERVK-ter	8412	2172
RMER17D	ERVK-ter	1154	466
RMER17D2	ERVK-ter	1562	715
RMER19A	ERVK-ter	2193	1060
RMER19B	ERVK-ter	5881	3104
RMER19C	ERVK-ter	4658	2063
RMER20A	ERVK-ter	1684	490
RMER20B	ERVK-ter	3460	967
RMER4A	ERVK-ter	2806	718
RMER4B	ERVK-ter	4989	1556
RMER6A	ERVK-ter	3964	1779
RMER6B	ERVK-ter	2006	594
RMER6C	ERVK-ter	4839	1935
RMER6D	ERVK-ter	2396	801
LTR16A	ERVL-ter	1500	316
LTR16C	ERVL-ter	1365	271
LTR33	ERVL-ter	1458	282
MLT2B1	ERVL-ter	1652	403
MLT2B2	ERVL-ter	1339	304
MLT2B3	ERVL-ter	1501	353
MLT2B4	ERVL-ter	1864	415
MLT2D	ERVL-ter	1562	336
MT2_Mm	ERVL-ter	2631	1182
MT2A	ERVL-ter	13600	3491
MT2B	ERVL-ter	16358	2415

Table S1 (continued)

LTRter (cont.)	Superfamily	Loci	base pair (x1000)
MT2B2	ERVL-ter	3945	737
MT2C_Mm	ERVL-ter	1980	711
RLTR28	ERVL-ter	2013	734
RMER10A	ERVL-ter	4433	1216
RMER10B	ERVL-ter	3291	780
RMER15	ERVL-ter	17626	3909
MLT1A	MaLR-ter	5027	956
MLT1A0	MaLR-ter	10480	2119
MLT1A1	MaLR-ter	9213	1399
MLT1B	MaLR-ter	9336	1904
MLT1C	MaLR-ter	7770	1691
MLT1D	MaLR-ter	8363	1839
MLT1E1A	MaLR-ter	1155	259
MLT1E2	MaLR-ter	1830	422
MLT1E3	MaLR-ter	1028	234
MLT1F	MaLR-ter	1550	375
MLT1F1	MaLR-ter	1606	343
MLT1F2	MaLR-ter	2291	562
MLT1G1	MaLR-ter	1073	234
MLT1G3	MaLR-ter	1067	237
MLT1H	MaLR-ter	2323	464
MLT1H2	MaLR-ter	1047	174
MLT1I	MaLR-ter	1792	303
MLT1J	MaLR-ter	2932	542
MLT1J1	MaLR-ter	1131	180
MLT1J2	MaLR-ter	1101	188
MLT1K	MaLR-ter	2798	509
MLT1L	MaLR-ter	1552	278
MTA_Mm	MaLR-ter	15289	5673
MTB	MaLR-ter	6377	2141
MTB_Mm	MaLR-ter	4708	1492
MTC	MaLR-ter	25369	7065
MTD	MaLR-ter	47854	12773
MTE2a	MaLR-ter	15178	3865
MTE2b	MaLR-ter	14407	3343
MTEa	MaLR-ter	21118	4563
MTEb	MaLR-ter	12110	2406
ORR1A0	MaLR-ter	2130	677
ORR1A1	MaLR-ter	3999	1153
ORR1A2	MaLR-ter	14289	4011
ORR1A3	MaLR-ter	4375	1141
ORR1A4	MaLR-ter	7739	2102
ORR1B1	MaLR-ter	14555	4272
ORR1B2	MaLR-ter	7995	2128
ORR1C1	MaLR-ter	7421	1965
ORR1C2	MaLR-ter	10665	2493
ORR1D1	MaLR-ter	20227	4856
ORR1D2	MaLR-ter	15000	3678
ORR1E	MaLR-ter	27796	6381

Table S2

		average piRNA coverage young	old	p-value
**MIWI2 early**	SINE	0.290756	0.112474	p < 2.2E-16
	LINE	0.374624	0.0563299	p < 2.2E-16
	LTRint	2.76772	0.642223	p < 2.2E-16
	LTRter	0.683309	0.140443	p < 2.2E-16
**MILI early**	SINE	0.202764	0.137685	p < 2.2E-16
	LINE	0.266554	0.0943798	p < 2.2E-16
	LTRint	1.33991	0.666733	p < 2.2E-16
	LTRter	0.54392	0.210231	p < 2.2E-16
**MILI late**	SINE	0.0933089	0.0617582	p < 2.2E-16
	LINE	0.11249	0.059659	p < 2.2E-16
	LTRint	0.869883	0.45142	p < 2.2E-16
	LTRter	0.269748	0.112719	p < 2.2E-16

P-values from Mann-Whitney U tests for the data in figure 2 (main text).
The populations of RTEs were divided in half according to millidivergence from consensus.
Young' loci constitute the lower half, 'old' the upper half.

Table S3

A) All RTE loci

		SINE	LINE	LTRint	LTRter
MIWI2 early	HIGH vs LOW	**4.12E-08**	**5.02E-09**	**2.17E-03**	**5.90E-09**
	HIGH vs MEDIUM	**2.35E-08**	**6.31E-13**	7.10E-02	**1.80E-10**
	MEDIUM vs LOW	1.00E+00	1.00E+00	9.77E-01	1.00E+00
MILI early	HIGH vs LOW	**4.45E-06**	**1.77E-08**	**3.09E-03**	**8.09E-08**
	HIGH vs MEDIUM	**1.32E-06**	**2.54E-11**	1.51E-01	**1.06E-08**
	MEDIUM vs LOW	1.00E+00	1.00E+00	9.21E-01	1.00E+00
MILI late	HIGH vs LOW	**6.14E-08**	**1.90E-08**	**2.11E-03**	**6.40E-09**
	HIGH vs MEDIUM	**1.11E-08**	**6.39E-14**	1.46E-01	**2.03E-11**
	MEDIUM vs LOW	1.00E+00	1.00E+00	8.62E-01	1.00E+00

B) Excluding young RTE loci

		SINE	LINE	LTRint	LTRter
MIWI2 early	HIGH vs LOW	**1.26E-07**	**3.09E-08**	**1.36E-03**	**4.53E-09**
	HIGH vs MEDIUM	**3.68E-06**	**5.45E-10**	2.37E-01	**1.12E-08**
	MEDIUM vs LOW	9.92E-01	1.00E+00	6.33E-01	1.00E+00
MILI early	HIGH vs LOW	**2.94E-07**	**3.48E-08**	**1.43E-03**	**5.01E-09**
	HIGH vs MEDIUM	**1.28E-05**	**9.14E-09**	2.89E-01	**1.65E-07**
	MEDIUM vs LOW	9.58E-01	1.00E+00	6.23E-01	9.71E-01
MILI late	HIGH vs LOW	**4.91E-09**	**2.15E-07**	**2.09E-03**	**1.13E-09**
	HIGH vs MEDIUM	**2.38E-09**	**2.49E-11**	3.33E-01	**3.68E-10**
	MEDIUM vs LOW	1.00E+00	1.00E+00	6.43E-01	1.00E+00

P-values from Mann-Whitney U tests for the data in figure 3 (main text).
Bonferroni corrected (n=36). Values below 0.05 highlighted in bold.
Excluding young elements were recorded without RTE loci with
millidivergences from consensus lower than 100.

Table S4

			MIWI2 early	MILI early	MILI late
		coverage			
SINE	young	inside	0.17784	0.25648	1.22070
		proximal	0.36099	0.42779	1.22555
		distal	0.19418	0.20124	0.89369
		p-values			
		inside/proximal	5.03E-08	7.19E-03	1.00E+00
		inside/distal	8.66E-01	1.00E+00	1.19E-04
		proximal/distal	2.51E-07	1.01E-07	5.03E-08
		coverage			
	median	inside	0.16203	0.20242	1.05552
		proximal	0.35479	0.43169	1.11432
		distal	0.19510	0.20682	0.73587
		p-values			
		inside/proximal	5.03E-08	2.16E-06	1.00E+00
		inside/distal	1.00E-01	1.00E+00	1.39E-04
		proximal/distal	5.03E-08	1.26E-06	1.51E-07
		coverage			
	old	inside	0.12216	0.15867	0.62842
		proximal	0.34238	0.41425	0.71482
		distal	0.15911	0.15583	0.39306
		p-values			
		inside/proximal	5.03E-08	5.03E-08	9.81E-01
		inside/distal	1.00E+00	1.00E+00	6.45E-04
		proximal/distal	3.79E-04	1.51E-07	7.46E-05

			MIWI2 early	MILI early	MILI late
		coverage			
LTRint	young	inside	1.81516	0.84874	0.71485
		proximal	2.14561	1.33308	0.53352
		distal	1.74681	0.83402	0.32245
		p-values			
		inside/proximal	4.91E-02	6.79E-04	1.00E+00
		inside/distal	1.00E+00	1.00E+00	1.39E-05
		proximal/distal	6.22E-04	1.96E-06	2.25E-06
		coverage			
	median	inside	1.13437	0.74348	0.63912
		proximal	1.96717	1.96989	0.87294
		distal	1.09512	0.67652	0.20050
		p-values			
		inside/proximal	1.86E-04	1.05E-03	1.00E+00
		inside/distal	1.00E+00	1.00E+00	5.20E-04
		proximal/distal	7.50E-05	6.07E-05	8.13E-08
		coverage			
	old	inside	1.15918	0.70773	0.74213
		proximal	1.60197	1.26597	0.71982
		distal	0.96600	0.56187	0.37383
		p-values			
		inside/proximal	7.01E-01	9.90E-01	1.00E+00
		inside/distal	9.95E-01	1.96E-01	7.41E-04
		proximal/distal	1.69E-04	1.13E-04	5.10E-06

			MIWI2 early	MILI early	MILI late
		coverage			
LINE	young	inside	0.13912	0.19449	0.12829
		proximal	0.29607	0.22224	0.09833
		distal	0.13039	0.09956	0.03179
		p-values			
		inside/proximal	1.46E-11	2.71E-07	9.87E-01
		inside/distal	1.00E+00	1.00E+00	1.99E-12
		proximal/distal	2.01E-11	4.15E-07	6.62E-12
		coverage			
	median	inside	0.13361	0.10222	0.09364
		proximal	0.32032	0.25780	0.10309
		distal	0.11382	0.09046	0.02498
		p-values			
		inside/proximal	5.40E-13	2.41E-10	1.00E+00
		inside/distal	5.85E-01	9.98E-01	1.20E-13
		proximal/distal	6.09E-12	3.54E-11	9.59E-14
		coverage			
	old	inside	0.12319	0.11985	0.10978
		proximal	0.29263	0.25882	0.07063
		distal	0.12124	0.11428	0.02376
		p-values			
		inside/proximal	2.12E-10	3.63E-08	1.00E+00
		inside/distal	1.00E+00	1.00E+00	8.79E-11
		proximal/distal	7.29E-11	5.51E-11	2.48E-10

			MIWI2 early	MILI early	MILI late
		coverage			
LTRter	young	inside	0.74649	0.59366	0.28293
		proximal	0.93930	0.77839	0.28874
		distal	0.72357	0.55778	0.15116
		p-values			
		inside/proximal	4.39E-11	4.86E-11	1.00E+00
		inside/distal	1.00E+00	1.00E+00	0.00E+00
		proximal/distal	0.00E+00	3.86E-12	0.00E+00
		coverage			
	median	inside	0.65847	0.49752	0.27672
		proximal	0.93005	0.82843	0.27029
		distal	0.60288	0.47198	0.12757
		p-values			
		inside/proximal	4.51E-12	1.08E-13	1.00E+00
		inside/distal	3.53E-01	9.87E-01	0.00E+00
		proximal/distal	0.00E+00	0.00E+00	0.00E+00
		coverage			
	old	inside	0.66269	0.48785	0.27425
		proximal	0.86442	0.79394	0.26576
		distal	0.55262	0.42003	0.10599
		p-values			
		inside/proximal	2.68E-07	1.49E-09	1.00E+00
		inside/distal	3.02E-02	3.76E-01	0.00E+00
		proximal/distal	0.00E+00	4.80E-14	0.00E+00

p<0.05
p<0.001
